# Feasibility of the new dry taste assessment method TASENS in older adults: a pilot study in two European countries

**DOI:** 10.1186/s40795-026-01381-6

**Published:** 2026-06-02

**Authors:** Vít Blanař, Jan Pospíchal, Doris Eglseer, Marie Chrástecká, Pavlína Brothánková, Jan Vodička, Silvia Bauer

**Affiliations:** 1 Faculty of Health Studies, University of Pardubice, Průmyslová 395, Pardubice, 532 10 Czech Republic; 2 Department of Otorhinolaryngology and Head and Neck Surgery, Pardubice Hospital, Kyjevská 44, Pardubice, 53203 Czech Republic; 3 Institute of Nursing and Health Sciences, Medical University of Graz, Neue Stiftingtalstraße 6, Graz, 8010 Austria

**Keywords:** Malnutrition, Older adults, Dry taste assessment method, TASENS, Age-related taste alterations, Feasibility, Pilot feasibility study

## Abstract

**Background:**

Despite existing studies, there is no consensus on standardised methods for taste assessment, and existing liquid methods have not been sufficiently proven to be accurate for older adults. Therefore, the study aims to conduct a pilot study on the feasibility of the new dry taste assessment method (TASENS) in older hospital patients and to gain initial insight into the association between taste alterations and malnutrition.

**Methods:**

A quantitative cross-sectional pilot study was carried out. The pilot study was conducted with 54 hospital patients from the Czech Republic and Austria with a median age of 77 years. The TASENS method was used to assess taste alterations, and nutritional status was evaluated using the MNA-SF. Feasibility was assessed using a 5-point Likert scale. The study adheres to the STROBE guidelines.

**Results:**

All participants found the duration of the assessment acceptable and the instructions clear, with the procedure being easy for most (96.3%). In Austria, 68% agreed that the assessment was practical, while in the Czech Republic, 48.3% agreed. Patients correctly identified an average of 68.3% of the tastes. The rate of correctly identified tastes was highest in participants with normal nutritional status (AT 67.6%; CZ 73.6%) and lowest in malnourished patients (AT 58.3%; CZ 65.6%).

**Conclusion:**

This pilot study identified the TASENS method to be a feasible tool for assessing taste alterations in older populations. Based on our data, approximately one-third of the patients had taste alterations, and people who had taste alterations were also more likely to have malnutrition.

**Supplementary Information:**

The online version contains supplementary material available at 10.1186/s40795-026-01381-6.

## Introduction

Ageing stands out as one of the crucial factors that influence taste perception [1]. Fluitman [2] suggested that a reduced number and function of taste buds, decreased saliva production, reduced neural response to taste stimuli, and impaired ability to fully masticate food contribute to a worsening of perception of taste. Additionally, dysfunction in retro-nasal olfaction may play a significant role. These changes can be worsened by general poor health, polypharmacy, metabolic systemic diseases, or neurological disorders. This may lead to manifest taste alterations, which are characterised by deviations from normal gustatory function, resulting in a reduced ability to taste (hypogeusia), a distortion of taste (dysgeusia), as well as the total lack of taste (ageusia) [3, 4]. Similar to presbycusis, the age-related increase in auditory thresholds, there is likely an age-related increase in the threshold for taste perception, requiring stronger stimuli to evoke taste sensations.

Although taste alterations are not typically considered life-threatening, they can significantly impact an individual’s nutritional status and overall quality of life, as well as their sense of joy in life’s experiences, thereby influencing the overall health of older adults. Taste alterations can lead to decreased appetite, changes in eating habits, undesired preferences for certain foods (e.g., overly sweet dishes), or unwanted alterations in food preparation (e.g., excessive salt or fat intake and/or inadequate protein intake) [2]. For example, the standard seasoning of meat in European and North American cuisine is predominantly salty. Patients with a diminished perception of salty taste may have a reduced inclination to consume meat, leading to a decreased protein intake. Fluitman [2] also found that patients with poor taste had a lower protein intake than patients with normal taste. Another study described that taste and smell disorders lead to reduced intake of vegetables and meat [5]. In the long run, low protein intake negatively affects muscles and wound healing, both of which pose significant risks in older adults [6, 7]. In line with this, a recent systematic review identified an association between taste disorders and sarcopenia [8]. Based on the studies mentioned above [2, 5–8], it can be assumed that there is a clear theoretical link between taste disorders, reduced food intake, risk of malnutrition, unbalanced macro and micronutrient intake and weight loss.

The prevalence of taste alterations was found to be 19% in community-dwelling adults (aged 40+) [4, 9] and 27% in older adult populations [4]. In patients with cancer, it is even higher, and rates of up to 86% are reported [10–12]. However, due to the huge heterogeneity in applied methods to assess taste alterations and a lack of consensus within the scientific community, it has not been possible to adequately compare the results of individual studies.

A widely used method for taste assessment is the use of taste strips by Burghart Messtechnik GmbH, Germany, first validated by Mueller et al. [13]. Alternative methods for taste assessment include liquid solutions [14, 15], whole-mouth methods using pump-sprays [16], and the application of different tastes using a cotton swab applicator [17]. One main challenge is the inability to precisely quantify the amount of taste solutions used in the studies. The lack of standardisation applied in liquid solutions or cotton swab applicators limits these methods. For example, in cotton swabs, the amount of taste solution absorbed into this material depends on the duration of soaking. In contrast, the amount of liquid released onto the tongue depends on the pressure applied to the tongue and the duration of application. Furthermore, the commonly applied methods do not allow a separate assessment of the taste perception in different areas of the tongue.

As a result, and to overcome the barriers of the already available methods, a new dry method of taste assessment (TASENS) was developed and tested in patients with Covid-19 at the University of Pardubice in the Czech Republic (CZ) [18]. The TASENS method is protected by patent no. CZ 310,140 B6 issued by the Industrial Property Office of the Czech Republic [19]. The main contribution of the newly developed method is its ability to overcome all of the above-mentioned barriers. TASENS uses a precisely defined quantity of a dried taste substance on each foil, which facilitates standardisation and thus comparability. Furthermore, TASENS allows a separate assessment of the taste perception on the right, left, or both sides of the tongue and uses two concentrations, which give hints at recognition thresholds and the degree of the taste alteration. The first results among Covid-19 patients and patients after ear surgery are promising and showed that TASENS can detect taste alterations correctly [19].

The aforementioned taste alterations, with their negative consequences on nutrition, can culminate in lower nutritional intake and subsequently malnutrition, which is defined as a condition resulting from inadequate nutritional intake or absorption, leading to altered body composition (decreased fat-free mass and body cell mass, resulting in decreased physical and mental function and impaired clinical outcomes of diseases [6]. Malnutrition is a highly prevalent disease among older individuals, affecting up to 28.0% patients in hospitals, 17.5% in nursing homes, and 8.5% in community settings [20]. There are already some studies available that describe the complex association of taste alteration and malnutrition [21, 22]. Nevertheless, the conducted studies are very heterogeneous and limited due to the usage of different methods to assess taste alterations.

In conclusion, taste changes are a relevant problem in older adults that need to be properly assessed to allow adequate identification and subsequent treatment. The new TASENS method allows the standardised and detailed assessment of taste perception, but the feasibility of this method has not yet been investigated in older adults. Knowledge about the feasibility in the older adult group is necessary before this assessment method can be spread in large-scale studies. In older populations, the feasibility of assessment methods is a crucial methodological consideration due to age-related cognitive, sensory, and physical limitations, which may influence test acceptability and validity. Furthermore, there has been limited knowledge on the association of taste alterations and malnutrition in older adults, and the heterogeneity in contemporary assessment methods limits comparability. Therefore, the research gap we aim to fill is to investigate the feasibility of the new TASENS method for assessing taste in older patients and to gain insight into the association of taste alterations in relation to malnutrition. This insight would allow a high-risk group to be identified early on and would enable early prevention of the negative consequences.

## Aims

The objective of this pilot study is to test the feasibility of the new dry taste assessment method (TASENS) in older hospitalised adults and to gain initial insight into taste alterations and malnutrition.

## Methods

### Design

A quantitative cross-sectional pilot study was carried out to test the feasibility of the new taste assessment method (TASENS) in older adults.

### Sample and setting

Data collection was conducted between October 2023 and February 2024 in the Czech Republic (CZ) and Austria (AT). Patients over 70 years of age in one Geriatric department of a District Hospital in the Czech Republic and the Department of Internal Medicine of a University Hospital in Austria were approached for participation in the research. Patients were enrolled in the study using a convenience sampling method. All eligible participants were invited by researchers to take part in the study during the period of data collection. We included only patients with written informed consent. The following exclusion criteria were applied:

- Age below 70 years.
- Cognitive status (MMSE < 24 points).
- Language barrier.
- Severe food allergy or allergy to plastics.
- Severe swallowing impairment.
- Having been treated for taste alteration already.
- Genetic taste alteration.
- Neurological, rheumatoid, or orthopaedic diseases that affect hand grip strength.
- Viral disease in the last 6 weeks (influenza, Covid-19, hepatitis, common cold).
- Ongoing radiotherapy or chemotherapy.
- Severe condition of ongoing disease (patient is not capable of participating).

### Data collection and measurement

Data collection was performed by nurses or nursing students who attended a training before the data collection in both countries. At the beginning of the data collection, the nurse checked the exclusion criteria and informed the patient about the whole data collection procedure. The patient was not allowed to eat, drink liquids other than clean non-sparkling water, smoke, chew gum, or brush his/her teeth for at least 1 h before the examination. The data collection was conducted in the patient’s room (if possible). During the assessment, a quiet environment was ensured, and potential distractions were minimised. Patients were examined in a seated or semi‑recumbent position in bed. Data collection was conducted in the morning or early afternoon, outside mealtimes, medication administration, and other routine medical or nursing procedures.

We collected data on general characteristics of the patients using a developed protocol for the data collection (Supplementary file 1), including their age, gender, medical diagnosis, condition of their teeth, and number/type of medications. Furthermore, a subjective assessment of taste and smell was performed in which the patients rated their taste and smell on a 10-point Likert scale (0 = worst; 10 = best). The condition of teeth was assessed using the question: “What is the condition of your teeth?” The patient’s response was verified by visual inspection of the patient’s oral cavity by the examiner before the taste examination.

Taste was assessed with the TASENS method [19]. The test consists of 24 steps during which the patient evaluates the four taste qualities (salt, sweet, sour, bitter). The taste qualities are either on the right, the left, or on both sides of the test foils and in two different concentrations (high and low concentration of the taste). The foils were made from the biocompatible polyethylene terephthalate film. Clean non-sparkling water was prepared for the patient to drink or rinse the mouth after every test. The instructions were given orally by the researcher. If the patient deviated from the standard procedure, they were corrected by the researcher.

At each step of the self-assessment, the patient places a test foil in his/her mouth. The examination was carried out in a standardised order of test foils. The foil was placed on the tongue up to the mark, so that the flavouring substance applied to the foil faces the surface of the tongue. While doing this, the patient points a finger to the selected answer (salt, sweet, sour, bitter) without removing the foil from the mouth. After selecting the answer, the patient takes the foil out of the mouth. The examiner then records the result for each foil in a standardised form. A correct answer was defined as the selection of the taste corresponding to the taste substance actually applied on the foil. A wrong answer was defined as the selection of a taste that did not correspond to the taste substance applied on the foil. No answer was defined as the inability of the respondent to select any taste. After each step, the patient rinses his mouth or drinks clean still water. The same procedure is followed until all 24 taste foils have been used. The number of correctly identified taste strips was counted for each taste category.

The nutritional status was assessed using the Mini Nutritional Assessment Short Form (MNA-SF), which is the short version of the MNA and consists of 6 questions. It is a commonly used screening tool to assess malnutrition risk in older individuals. All questions can receive between 0 and 2 points, whereas fewer points indicate a worse nutritional status. The values of the individual responses of the MNA-SF were summed. Scores between 12 and 14 indicate normal nutritional status; scores between 8 and 11 indicate a risk of malnutrition, and scores of ≤ 7 indicate malnutrition [23–25]. The MNA-SF completion included measurement of height and body weight of the patient in order to calculate BMI according to the formula: BMI = weight [kg]/height [m²]. Furthermore, muscle mass was assessed by calf circumference measurement and hand grip strength because muscle assessment is recommended by ESPEN guidelines as an important parameter in the evaluation of malnutrition [6]. Handgrip strength (in Kg) was assessed in a sitting position using a certified dynamometer. According to the recommendations from Steiber et al. [26], we performed a measurement on each hand, and the dominant hand was marked. Finally, the maximum value achieved with either hand is used as a summary measure of an individual’s isometric strength of the hand and forearm muscles. The reference values for the central European population are stratified by gender and age and were developed based on an analysis of 11,790 individuals [26].

The feasibility of the TASENS was evaluated on a 5-point Likert scale (fully agree to fully disagree). The patients answered questions about the duration, the instructions, and the procedure of the TASENS method. Furthermore, we recorded the duration of the TASENS method, including education prior to assessment, in minutes.

### Ethical considerations

The study complies with ethical principles described in the Declaration of Helsinki and was approved by the responsible ethical committees in the participating countries (CZ: Statement of the Ethics Committee for Pardubice and Svitavy Hospitals dated August 18, 2023; AT: Ethical Committee of Medical University of Graz, Statement number 35–481 ex 22/23). All participants received oral and written information about the study and had to give their written informed consent before participating in the study.

### Data analysis

The data obtained within this pilot study were analysed using IBM SPSS Statistics 28 for Windows [27]. Because this was a pilot study, the data analysis focused mainly on descriptive statistics to evaluate feasibility and to gain insight into taste and malnutrition. Descriptive analyses of all variables were conducted to determine data distribution and identify outliers. For continuous variables, medians, means, standard deviations, and interquartile ranges were calculated. For categorical variables, absolute and relative frequencies were used.

The feasibility of the TASENS method was assessed using items on a five-point Likert scale (strongly agree – strongly disagree), focused on test duration, clarity of instructions, difficulty, and practical aspects of the procedure. These items were analysed using descriptive statistics in the form of frequencies and percentage distributions of responses. The results of the feasibility assessment were also shown graphically using bar charts to allow clear visualisation of response distributions in individual categories and comparison between countries. Test duration was analysed as a continuous variable and described using the median and range.

Because of the sample size and the exploratory aim of the pilot study, no statistical tests or hypothesis testing were performed. The analysis was not focused on statistical significance, but on describing data characteristics and assessing the feasibility of the TASENS method in a clinical setting.

## Results

In total, 54 participants took part in the pilot study, 53.7% of whom were from the Czech Republic. The median age was 77 years; 74 years in AT and 80 years in CZ. In AT, the majority of the participants were male (72%), while in CZ, the majority were female (58.6%). The mean number of medications was 9, while it was 10 in AT and 9 in CZ. The most common disease in both countries was a circulatory system disease. This was followed by endocrine and genitourinary diseases in AT and diseases of the musculoskeletal system and skin in the CZ. In AT, most of the participants had a normal nutritional status (60.0%), and 36.0% had a risk of malnutrition according to MNA-SF. In the CZ, most of the participants had a risk of malnutrition (44.8%), and 41.4% had a normal nutritional status. Most of the participants had a dental prosthesis in both countries (55.6%). Damaged or ill teeth were more prevalent in the CZ (24.1%) than in AT (8.0%) (Table 1).

**Table 1:** General characteristics

	Austria (*n* = 25)	The Czech Republic (*n* = 29)	Total (*n* = 54)
Age median (IQR)	74 (72–80)	80 (74–82)	77 (73–82)
Female gender (n)	28.0% (7)	58.6% (17)	44.4% (24)
Smoking	-	10.3% (3)	5.6% (3)
Mean number of medications (SD)	10 (± 4)	9 (± 3)	9 (± 3)
Diseases of the circulatory system (n)	80.0% (20)	69.0% (20)	74.1 (40)
Diseases of the musculoskeletal system (n)	16.0% (4)	55.2% (16)	37.0% (20)
Skin disease (n)	4.0% (1)	55.2% (16)	31.5% (17)
Diabetes mellitus (n)	24.0% (6)	27.6% (8)	25.9% (14)
Endocrine, nutritional, and metabolic diseases (n)	44.0% (11)	3.4% (1)	22.2% (12)
Diseases of the genitourinary system (n)	32.0% (8)	3.4% (1)	16.7% (9)
BMI in kg/m^2^ mean (SD)	27.1 (5.6)	28.5 (5.7)	27.8 (5.7)
Hand grip strength maximum value (either hand)^1^ in kg mean (SD)	26 (8)	23 (7)	24 (8)
Calf circumference in cm median (IQR)^*^	35 (32–36)	36 (31–43)	35 (32–39)
MNA-SF			
Normal (n)	60.0% (15)	41.4% (12)	50.0% (27)
Risk of malnutrition (n)	36.0% (9)	44.8% (13)	40.7% (22)
Malnourished (n)	4.0% (1)	13.8% (4)	9.3% (5)
Condition of the teeth			
Normal (n)	28.0% (7)	13.8% (4)	20.4% (11)
Paradentosis (n)	-	10.3% (3)	5.6% 73)
Dental prosthesis (n)	64.0% (16)	48.3% (14)	55.6% (30)
Damaged/ill (n)	8.0% (2)	24.1% (7)	16.7% (9)
Other (n)	-	3.4% (1)	1.9% (1)
Dry mouth^±^ (n)	44.0% (11)	62.1% (18)	53.7% (29)

*IQR* Interquartile range, *SD* standard deviation, *MNA-SF* Mini Nutritional Assessment

^1^ Following published recommendations, the maximum value achieved with either hand is used as a summary measure of a person’s isometric strength of the hand and forearm muscles (Steiber 2016)

^*^Austria *n*=22; Czech Republic *n*=18; total *n*=40

^±^ Czech Republic *n*=28; Total *n*=53

The median of the subjective assessment of the senses of smell and taste of the participants, measured on a VAS (Visual Analogue Scale) from 0 (very bad) to 10 (best), was 8 in both countries. In AT, a problem with the taste of bitter was among the most prevalent (15.4%), while in the CZ, the taste of salty was problematic (13.8%). Only patients who subjectively experienced taste issues responded to the question about specific taste problems. In AT, 7.7% indicated to have no taste at all, and 23.1% mentioned that everything tastes different. None of the participants from the CZ indicated having no or a different taste (Table 2).

**Table 2:** Subjective assessment of the senses of smell and taste

	Austria (*n* = 25)	The Czech Republic (*n* = 29)	Total (*n* = 54)
Sense of smell (0–10 points) median (IQR)^*^	8 (7–9)	8 (8–10)	8 (7–10)
Sense of taste (0–10 points) median (IQR)	8 (7–10)	8 (8–10)	8 (8–10)
Problem with salty^#^	7.7% (1)	13.8% (4)	11.9% (5)
Problem with sour^#^	0%	0%	0%
Problem with bitter^#^	15.4% (2)	0%	4.8% (2)
Problem with sweet^#^	0%	3.4% (1)	2.4% (1)
No taste at all^#^	7.7% (1)	0%	2.4% (1)
Everything tastes different ^#^	23.1% (3)	0%	7.1% (3)

*Austria *n* = 24; Total *n* = 53

# Austria *n* = 13; Czech Republic *n* = 29; Total *n* = 42

In total, an average of 68.3% of the tastes were correctly identified, while this percentage was slightly higher among participants from the CZ (69.1%) compared to AT (67.2%). In both countries, sweet (AT 72.7%; CZ 78.7%) and bitter (AT 71.5%; CZ 73.0%) were among the most prevalent correctly identified tastes, while sour (AT 58.3%; CZ 61.5%) was the least often correctly identified taste. The correct identification of the taste was the easiest for AT participants when the taste was on both sides of the taste foil (72.3%). In the CZ, participants most often correctly rated taste strips with the solution distributed on the right side of the strip (72.1%). Taste strips with high concentrations were easier to correctly identify in the CZ (73.0%), but not in AT (67.4%). (Table 3).

**Table 3:** Objective taste assessment with TASENS (% of correct answers)

	Austria (*n* = 25)	The Czech Republic (*n* = 29)	Total (*n* = 54)
Total mean (SD)^*^	67.2% (16.2)	69.1% (16.8)	68.3% (16.4)
Salt mean (SD)^*^	66.7% (24.6)	63.2% (27.6)	64.7% (26.1)
Sweet mean (SD)	72.7% (20.3)	78.7% (22.2)	75.9% (21.4)
Bitter mean (SD)^#^	71.5% (24.8)	73.0% (25.4)	72.3% (24.9)
Sour mean (SD)^#^	58.3% (32.2)	61.5% (32.2)	60.1% (31.9)
Right side mean (SD)^#^	63.0% (23.4)	72.1% (18.3)	67.5% (21.0)
Left side mean (SD)^#^	65.1% (20.5)	66.4% (19.2)	65.8% (19.6)
Both sides mean (SD)^*^	72.3% (16.2)	69.1% (16.8)	70.9% (21.8)
Low concentration mean (SD)^*^	67.4% (17.2)	65.2% (18.6)	66.2% (17.9)
High concentration mean (SD)^*^	67.4% (19.5)	73.0% (19.8)	70.4% (19.7)

*Austria *n* = 23; Total *n* = 52

# Austria *n* = 24; Total *n* = 53

The rate of correctly identified tastes was highest among participants with normal nutritional status (AT 67.6%; CZ 73.6%) and lowest among malnourished participants (AT 58.3%; CZ 65.6%) (Table 4).

**Table 4:** Association of taste (in % of correct answers in TASENS) and malnutrition (according to MNA-SF)

	Austria (*n* = 23)	The Czech Republic (*n* = 29)	Total (*n* = 52)
Normal nutritional status mean (SD)	67.6% (20.0)	73.6% (19.7)	70.4% (19.7)
Risk of malnutrition mean (SD)	67.7% (7.9)	66.0% (15.1)	66.7% (12.6)
Malnourished mean (SD)	58.3%	65.6% (12.9)	64.2% (11.6)

*SD* Standard Deviation

**Fig. 1:**
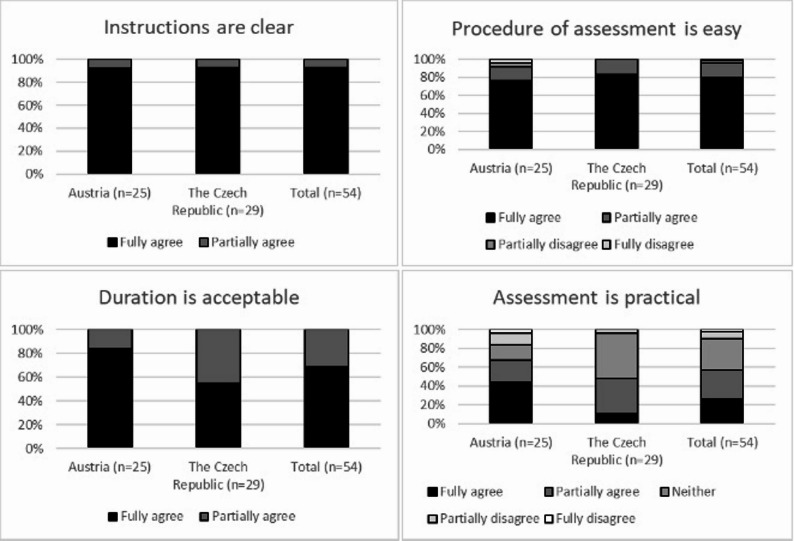
Feasibility of TASENS

The duration of the assessment with TASENS took a median of 20 min in AT and 11 min in the CZ. All participants in both countries either fully or partially agreed that the duration of the assessment is acceptable and that the instructions are clear. The procedure of assessment was easy for most of the participants (79.6%). In AT, most participants fully (44.0%) or partially agreed (24.0%) that the assessment is practical, and 16% partially or fully disagreed with this statement. In the CZ, most of the participants (48.3%) neither agreed nor disagreed, followed by 37.9% of the CZ participants who partially agreed that the assessment is practical (Fig. 1).

## Discussion

This study aimed to test the feasibility of the new dry taste assessment method (TASENS) in older hospitalised adults. The TASENS method revealed that 69.1% of the taste stimulants were identified correctly by patients in the Czech Republic and 67.2% of the Austrian patients. In both countries, sweet and bitter were among the most prevalent correctly identified tastes, while sour was the least often correctly identified taste. The rate of correctly identified tastes was the highest among participants with normal nutritional status and lowest among malnourished participants. Most of the participants from both countries fully or partially agreed that the assessment with the TASENS is acceptable, clear, and easy. Only the practicability was rated worse, especially in the Czech Republic (48.2% in CZ agree that the method is practical).

Regarding the general characteristics of the research sample, patients from the Czech Republic were older, had poorer nutritional status, and worse dental health compared to those from Austria. Additionally, more women were present in the Czech sample, with gender known to influence taste perception [28]. Nutritionally, more Czech patients were at risk of malnutrition (44.8%) compared to Austria, where most had a normal status (60%). Dental health was also worse in the Czech Republic, with 24.1% having damaged teeth versus 28.0% with normal teeth in Austria. Consequently, results from each country should be considered separately.

### Taste alterations

The objective taste assessment using the TASENS method revealed that two-thirds of taste stimulants were identified correctly, while one third was not. This is consistent with Rawal [4], who found that 27% of older patients had taste alterations. In both countries, sour and salty were the least often correctly identified tastes. This underlines the results of the literature that found that thresholds for salty, bitter, sour, and umami tastes are significantly higher among older adults than in younger adults [29], while usually the sensitivity to sweetness is maintained.

Next to the objective assessment, we also applied subjective assessment methods to identify taste alterations and found only a limited association between these two types of assessment methods. Patients rated their taste in the subjective assessment (using a Visual Analogue Scale from worst 0 to best 10) “better” than it was rated in the objective assessment with TASENS. The median subjective taste rating by participants was 8 (IQR 8–10), which can be regarded as quite good, whereas the average success rate in the objective taste assessment was 68%. Nevertheless, results agree that the salty taste was the most problematic one (in the objective as well as in the subjective assessment). Additionally, the subjective assessment revealed that patients were complaining about the fact that all foods tasted different than what they were accustomed to. This may also be related to the alteration of retro-nasal olfaction [30].

Previous studies also show that subjective and objective taste assessments differ. The authors conclude that the primary health condition of the patients may play a role. Patients in better health conditions are more likely to capture possible taste disorders subjectively compared to patients in worse health conditions. This was shown by Park et al. [31], who displayed a higher correlation between objective and subjective assessment in healthy controls compared to patients with burning mouth syndrome. Another explanation for this discrepancy may be the fact that taste alterations can develop over years to decades at a slow pace, which patients may hardly notice, or they may automatically adjust the flavour of their food. Additionally, taste is a sense whose changes are not perceived with the same intensity and subjective significance, for instance, vision or hearing [32].

Nevertheless, the discrepancies observed in our sample between objective and subjective taste assessment highlight the significant importance of this issue in older adult patients, where this problem has not been sufficiently described and explored. Some patients in the sample also rated their taste as poor without being able to articulate the specifics. Especially in Austria, many participants indicated having taste problems, but were unable to differentiate the problems. This further underlines the importance of objective assessment.

Our results show that the rate of correctly identified tastes was highest among participants with normal nutritional status and lowest among malnourished participants, which gives hints at a positive association between nutritional status and taste alterations. Other studies also highlight the association between taste disorders and, more importantly, undesirable changes in eating habits, which can lead to changes in body composition [33, 2, 21, 22]. However, due to their heterogeneity and the inability to compare and quantify the results of the methods used, it remains unclear how significant a role taste alteration plays in the nutrition of older adults. Based on our results and recent scoping review, the taste alterations represent an important element within the complex sensory–nutritional pathway linking age-related sensory decline to altered food intake, malnutrition, and functional impairment in older adults [34]. Our results, together with current relevant literature, support the idea of the conceptual understanding of the pathway between ageing, taste changes, appetite and food intake, nutrition, muscle function, sarcopenia, and clinical outcomes. Nevertheless, data from a larger study are necessary to provide a reliable conclusion.

### Feasibility of TASENS

The feasibility of the TASENS was rated as good. In most cases, patients reported that the instructions for conducting the assessment given by the nurse/researcher were clear and that the entire process was easy to perform. The duration of the TASENS test to some extent depends on the overall condition of the patient and their ability to cooperate. An interesting difference in the duration of the assessment was observed between patients from the Czech Republic and Austria. The TASENS assessment took a median of 20 min in Austria and 11 min in the Czech Republic. Despite the older age of the patients in the Czech Republic, their self-assessment time was shorter. One reason for this could be the length of patient education before the assessment, which was included in the total assessment time. Researchers in Austria have spent more time explaining the importance of the taste assessment to patients.

Overall, the positive evaluation of the feasibility of the TASENS method was surprising, given the long mean assessment time of an average of 15 min. However, since the practicability was considered a problem, one of the further research objectives is to evaluate the length of the TASENS test in a larger research sample from multiple hospitals and across different patient settings, to shorten the TASENS method. One way to increase patient compliance and improve their perception of the practical usability of TASENS could be more thorough education before the assessment. The duration of taste assessment using other methods is not typically published. However, it can be assumed that the time required corresponds to the number of individual taste substances and their concentrations. For example, the taste test with paper strips includes 36 strips for testing [35], the Taste Disc involves 26 discs for testing [36], and the Taste Strips (Burghart Messtechnik GmbH) include 18 strips [2], as compared to 24 taste strips using the TASENS method.

To a limited extent, in addition to the TASENS dry testing method, the literature also describes similar methods that use different principles for applying dry taste substances in the oral cavity. These include the “Edible Taste Film” [37] and the “Dried Taste Strips Test” [38]. The advantage of the TASENS concept is that the taste substance cannot spread to the palate or to other parts of the tongue that are not targeted by examination. However, these three methods have not yet been compared with each other in any research study. Therefore, ease of use, feasibility, and performance have not yet been investigated.

### Limitations

As this was a pilot study with a limited number of participants, the results cannot be generalised. For this reason, no significance testing was performed, as the study did not have sufficient statistical power for generalisation. The primary aim of the pilot study was to obtain initial insights and to explore the characteristics and applicability of the new research method (TASENS), rather than to confirm hypotheses or produce definitive conclusions. Nevertheless, the findings provide valuable information regarding the feasibility of the TASENS method and will serve as a basis for the design and implementation of a subsequent large-scale study.

## Conclusion

To better understand the complex relationship between taste and malnutrition in older individuals, comprehensive and standardised approaches are needed to assess age-related changes in taste. An important step may be the further development and evaluation of new methods, such as TASENS, aimed at improving the quantification of taste alterations. In our pilot study, the TASENS method indicates preliminary feasibility within this pilot sample, which was assessed by evaluation of instructions, time duration, practicality of assessment and how easy it was to perform the assessment. Patients generally reported that the instructions provided by the nurse/researcher were clear and that the TASENS test was easy to perform, with the duration of the test depending on the instructions given and the patient’s overall condition and cooperation. Notably, despite the test’s duration, it was deemed acceptable by patients, with differences observed between those in the Czech Republic and Austria. The high discrepancy between subjective taste perception and objective measurement of taste alterations emphasises the need for standardised and validated tools. Therefore, the objective assessment of taste alterations with feasible and standardised methods may be beneficial. Health care professionals should be aware of the relevance of taste alterations and recognise early signs, e.g., an excessive preference for certain tastes, early on. These efforts may help to prevent the deterioration of taste as well as the negative consequences of impaired taste on nutritional status. These findings should be interpreted cautiously, given the pilot nature of the study.

## Supplementary Information

Supplementary Material 1.

## Data Availability

The datasets used and/or analysed during the current study are available from the corresponding author on reasonable request.

## Abbreviations

AT: Austria
BMI: Body Mass Index
CZ: Czech Republic
ESPEN: European Society for Clinical Nutrition and Metabolism
IQR: Interquartile Range
MNA: Mini Nutritional Assessment
MNA‑SF: Mini Nutritional Assessment – Short Form
MMSE: Mini-Mental State Examination
NHANES: National Health and Nutrition Examination Survey
ORL: Otorhinolaryngology
SD: Standard Deviation
STROBE: Strengthening the Reporting of Observational Studies in Epidemiology
TASENS: Taste Assessment Sensoric Method
VAS: Visual Analogue Scale
