# Supplementary material for: Feasibility of the new dry taste assessment method TASENS in older adults: a pilot study in two European countries
Source: BMC Nutr. 2026 Jun 2;12:158. doi: 10.1186/s40795-026-01381-6 (PMC13465136; doi:10.1186/s40795-026-01381-6)
Supplement: Supplementary file 1 [file 40795_2026_1381_MOESM1_ESM.docx]

**Supplementary file - Data collection protocol for pilot testing (96p11)**

**Exclusion criteria**

- Severe food allergy or allergy to plastics
- Severe swallowing impairment
- Patients who are in treatment of taste assessment already, genetic taste disorders
- Patients with neurological, rheumatoid or orthopedics diseases that affects hand grip strength
- Patients younger than 70 years
- Dementia
- Language barrier
- Inability to measure weight and height
- Patients who smoke, chew gum, eat, or drink anything other than water in the last hour preceding the taste test
- Viral disease in the last 6 weeks (influenza, covid, hepatitis, common cold)
- Ongoing radio- or chemotherapy
- Severe condition of ongoing disease (patient is not capable to participate)

**Questions**

***General characteristics***

1. Age (in years)
2. Sex
   - male
   - female
   - client prefers not to say
3. Which medical diagnoses do the patient have?
   - Diseases of the circulatory system (not stroke)
   - CVA/stroke
   - Diseases of the musculoskeletal system
   - Diseases of the digestive system
   - Respiratory disease
   - Diseases of the nervous system (including spinal cord lesions/paraplegia)
   - Diseases of the eye/adnexa or ear/mastoid process
   - Endocrine, nutritional and metabolic diseases (not diabetes mellitus)
   - Diabetes mellitus
   - Neoplasms
   - Diseases of the blood and blood-forming organs and disorders involving the immune mechanism
   - Mental and behavioural disorders including dementia
   - Diseases of the genitourinary system
   - Infectious and parasitic diseases
   - Diseases of the skin and subcutaneous tissue
   - Other
   - Unknown/no diagnosis
4. Do you smoke?
   - No
   - Yes
5. Number of medications the patient takes per day:
   - ---------------------------------------
6. Do you take these kinds of drugs and if yes, which one?
   - Antimycotics
   - Antibiotics
   - Anti-inflammatory drugs
   - Immunosuppressants
   - Neurological medications
7. Do you have or had a facial nerve palsy?
   - No
   - Yes
8. Do you have or had oncological disease of the head and neck?
   - No
   - Yes
9. Do you had a middle ear surgery?
   - No
   - Yes

***Nutritional status***

MNA-SF

***Handgrip strength***

Dynamometer

***Taste***

1. Rate your smell on the scale

**0,____,____,____,____,____,____,____,____,____,____,10**

Worse	Best

2. Rate your taste on the scale

**0,____,____,____,____,____,____,____,____,____,____,10**

Worse	Best

3. If you have a taste disorder (7or less): With which flavor do you have problems? (multiple choice)
   - Sweet
   - Salt
   - Sour
   - Biter
   - I have no taste at all
   - Food tastes different than it should
4. Did you have a taste disorder after the illness of COVID 19?
   - No, I did not have COVID-19
   - No, I had COVID-19 but no taste disorder because of COVID-19
   - Yes, I had COVID-19
   - Yes, I had COVID-19 and I have a taste disorder because of COVID-19
5. Do you have a bad taste (aftertaste) in your mouth or tongue?
   - No
   - Yes
6. Have you ever been in treatment for a taste and/or smell disorder?
   - No
   - Yes
7. Do you have a smell disorder?
   - No
   - Yes
8. Do you feel dry in your mouth?
   - No
   - Yes
9. What is the condition of your teeth?
   - Normal
   - Paradentosis (periodontal disease)
   - Dental prosthesis
   - Damaged/ill teeth’s
   - Other

***Taste assessment***

TASENS – standard testing
